# Primary Hepatic Gastrinoma Causing Zollinger-Ellison Syndrome: A Rare and Challenging Diagnosis

**DOI:** 10.3390/cancers4010130

**Published:** 2012-02-14

**Authors:** Adrian Harvey, Janice L. Pasieka, Hassan Al-Bisher, Elijah Dixon

**Affiliations:** Division of General Surgery and Surgical Oncology, Department of Surgery and Oncology, University of Calgary, Calgary, Alberta T2N 2T9, Canada; E-Mails: adrain.harvey@albertahealthservices.ca (A.H.); dr.bisherhassan@yahoo.com (H.A.-B.); elijah.dixon@albertahealthservices.ca (E.D.)

**Keywords:** gastrinoma, primary hepatic gastrinoma, Zollinger-Ellison syndrome

## Abstract

The majority of gastrinomas causing Zollinger-Ellison syndrome (ZES) are located in the duodenum or the pancreas. Primary hepatic gastrinomas (PHG) are extremely rare and difficult to diagnose because the liver is the commonest site of metastatic disease and gastrinomas can be very small. Furthermore, gastrinomas are typically slow-growing thus a missed, occult primary tumour may not become evident for many years. The diagnosis of PHG is therefore dependent on a careful search for a primary and long-term biochemical follow-up following curative hepatic resection. We report a case of a 7 cm PHG in a 48 year old man with ZES. Preoperatively, both a basal and stimulated gastrin levels were elevated. Surgical exploration including intraoperative ultrasound and duodenotomy, failed to reveal a primary. Patient underwent a right hepatectomy. Yearly, gastrin and secretin stimulation tests remain normal 6 years following surgery. He remains symptom free off all medication. An additional 26 cases of PHG were found. Including this case, 21 had at least 1 year follow-up, however only eight had greater than 5 years (median 24 months). Post-op gastrin levels were reported in 25, however provocative testing was done in only 10. Persistence and recurrence occurred in one and four, respectively. PHG causing ZES is extremely rare. Although the current literature claims to include 26 additional cases of PHG, without a thorough search for the primary and long-term follow-up data including provocative testing, this diagnosis remains a challenge.

## 1. Introduction

Gastrinomas account for 20% of functioning neuroendocrine tumors (NET) of the pancreas [1]. Excessive gastrin secretion from these tumors is responsible for the clinical syndrome described by Zollinger and Ellison in 1955 [2]. Zollinger/Ellison syndrome (ZES) is characterized by gastric acid hypersecretion resulting in severe acid-related peptic ulcer disease and secretory diarrhea. In approximately 25% of cases, gastrinomas occur in the context of the familial disorder, multiple endocrine neoplasia type 1 (MEN 1). The distinction between familial and sporadic disease is an important one that carries significant implication for treatment and follow-up.

Primary gastrinomas are typically located in the duodenum or pancreas. More than 90% of primary tumors are found in the so-called “gastrinoma triangle” an area bounded by the neck of the pancreas, the confluence of the cystic and common bile ducts and the junction of the second and third portions of the duodenum [1]. A small percentage of primary tumours (5%–6%), are located outside this area. Gastrinomas in atypical locations have been reported in lymph nodes, the biliary tree, ovary, kidney, heart, stomach, jejunum, the omentum and the liver [3,4,5]. Making a firm diagnosis of a primary gastrinoma in sites such as the liver and/or lymph nodes is problematic given that these are the typical locations of metastatic disease and the primary tumours are often too small to be detected using standard pre-operative testing. Over long-term follow-up, a significant portion of previously diagnosed “primary lymph node” gastrinomas has been shown to be metastatic disease once the primary tumour becomes apparent [5].

As is the case with primary lymph node gastrinoma, primary hepatic gastrinomas (PHG) are extremely rare and difficult to diagnose because the liver is also a common site of metastatic disease. To date only 26 cases of apparently PHG have been reported in the English language literature (Table 1) [3,4,6,7,8,9,10,11,12,13,14,15,16,17,18,19,20,21,22,23,24,25,26,27]. All of these cases were confirmed histopathologically and 23 had documented preoperative biochemical evidence of ZES. However, not all of these underwent a “complete” operative exploration for a primary tumour and many of them have only reported relatively short-term follow-up.

**Table 1:** Presentation and follow-up of the primary hepatic gastrinomas in the literature.

Case No./Reference	*Presentation*			*Localization*		Follow-up				Recurrence	Persistance
	Age	Clinical ZES	FSG	Pre-op	Intra-op	Immediate Post-op		Long-Term			
						FSG	Provacative test	Length (months)	5 Years		
#1/[10]	44	√	√	√	IOUS Palp	√	√	12	x	x	x
#2/[9]	46	√	√	√	IOUS Palp Bx	√	x	2	x	x	x
#3/[7]	27	√	√	√	Palp	√	x	42	x	x	x
#4/[15]	13	√	√	√	“Careful search”	√	x	24	x	x	x
#5/[21]	50	√	√	√	IOUS TI	√	x	18	x	x	x
#6/[18]	46 *	√	√	√	IOUS	√	√	>96	√	x	x
#7/[18]	46 *	√	√	√	IOUS	√	√	>96	√	x	x
#8/[18]	46 *	√	√	√	IOUS	√	√	72	√	√	x
#9/[12]	57	√	√	√	Palp	√	√	6	x	x	x
#10/[16]	39	√	√	√	IOUS Whipple	√	x	12	x	x	x
#11/[19]	51	√	√	√	X	√	x	2	x	x	x
#12/[20]	50	√	√	√	IOUS SVS	√	√	60	√	√	x
#13/[8]	13	√	√	√	Palp Bx	√	x	12	x	√	x
#14/[13]	9	√	√	√	x	√	x	36	x	-	√
#15/[3]	57	√	√	x	Palp	√	x	14	x	x	x
#16/[27]	30	√	√	√	Palp	√	x	136	√	x	x
#17/[26]	8	√	√	√	Palp	√	x	18	x	x	x
#18/[24]	83	x	x	-	-	x	x	x	x	-	-
#19/[25]	29	√	√	√	IOUS TI	√	x	36	x	x	x
#20/[22]	56	√	√	√	IOUS Palp	√	x	20	x	x	x
#21/[11]	9	√	√	√	x	√	x	6	x	x	x
#22/[14]	39	√	√	√	IOUS, Palp SVS	√	√	240	√	√	x
#23/[23]	61	√	√	x	Palp	√	x	1	x	x	x
#24/[17]	50	√	x	√	Palp	√	√	24	x	x	x
#25/[6]	49	x	x	x	x	x	x	69	√	x	x
#26/[4]	23	√	√	√	Palp	√	√	24	x	x	x

*: mean age; Palp: palpation of the duodenum and pancreas; IOUS: intra-operative ultrasound; TI: endoscopic trans-illumination; Bx: lymph node biopsies; X: not reported; √: confirmed in the case report.

In this paper, we report a case of primary hepatic gastrinoma that underwent curative hepatic resection with no evidence of clinical, biochemical or radiological recurrence at the 6th year follow-up. We carefully scrutinized the work-up, treatment and follow-up of the additional 26 cases reported in the literature and challenge the diagnosis of PHG in some of these.

## 2. Case Report

**Figure 1:**
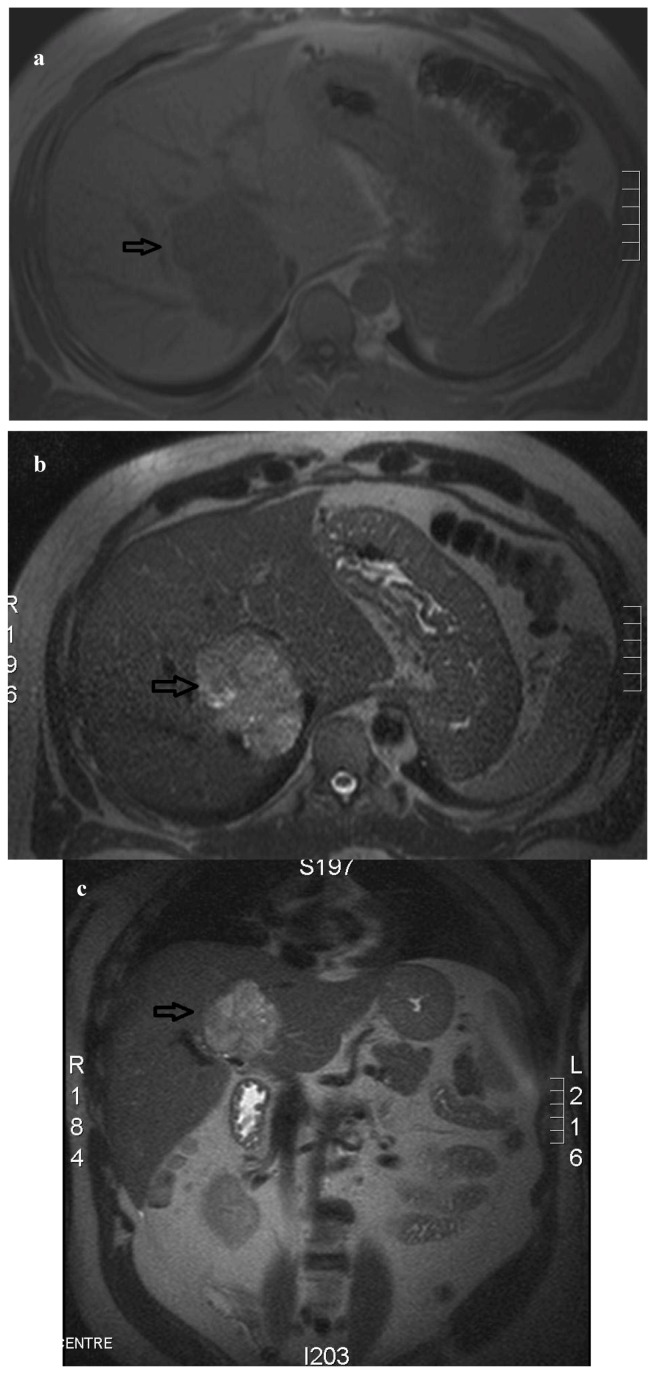
(**a**) An axial T1-weighted MRI of the abdomen demonstrates a solitary lesion (arrow) in the right lobe of the liver, axial. (**b**) T2-weighted MRI shows a single hyperintense liver lesion (arrow). (**c**) T2-weighted MRI coronal views.

A 48-year-old male presented with a 2-year history of intermittent diarrhea and vague abdominal pain. The patient described a long-standing problem with significant heartburn and reflux. His symptoms had become more frequent and severe in the few months prior to presentation. He described a weight loss of approximately 10 kilograms over the same time period. Physical exam reveal no abnormality. A colonoscopy, barium enema and small bowel follow-through all were negative. He underwent an upper GI endoscopy, which demonstrated significant gastritis and multiple duodenal ulcers. His fasting serum gastrin (FSG) level after stopping all anti-secretory medications was moderately elevated at 288 pg/mL (normal < 100 pg/mL) and Chromogranin A level was also elevated at 340 ng/mL (normal < 34 ng/mL). Both enhanced computerized tomography (CT) and magnetic resonate imaging (MRI) scans demonstrated a solitary 7.0 × 5.7 cm lesion within the right lobe of the liver (Figure 1).

**Figure 2:**
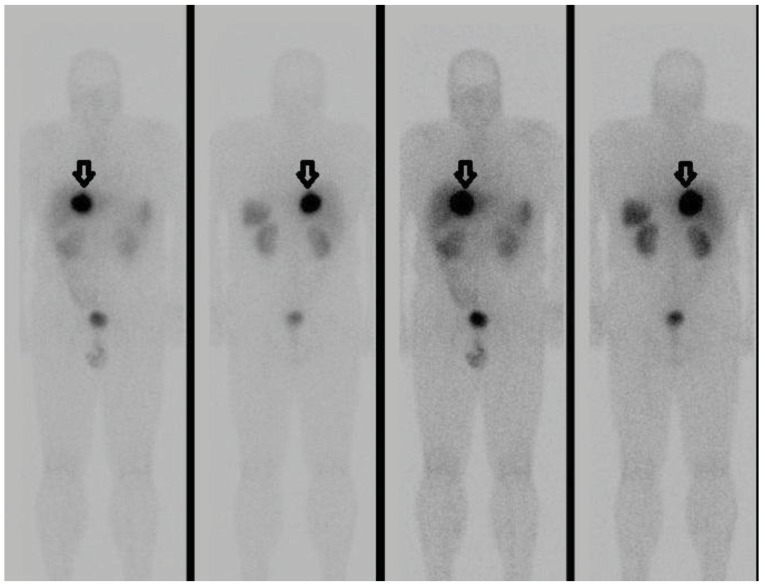
Anterior and posterior octreotide whole body scans show intense uptake of the somatostatin analogue by the solitary liver lesion (arrow) and no evidence of uptake outside the liver.

An image-guided core biopsy was consistent with a neuroendocrine tumour. Whole body octreotide scan and SPECT showed a solitary liver mass with no evidence of disease outside the liver (Figure 2).

At laparotomy, the pancreas was fully mobilized to allow for careful palpation and intra-operative ultrasound (IOUS) assessment. A duodenotomy was performed and the duodenal mucosa was carefully palpated from the pylorus to its fourth portion. A regional lymph node dissection failed to identify any primary or regional disease. An extended right hepatic lobectomy and cholecystectomy was performed. The patient’s post-operative course was unremarkable and he was discharge on day seven. Pathology revealed a 6.5 × 6.0 × 5.0 cm neuroendocrine tumour that stain positive for gastrin, Chromogranin A and synaptophysin. All of the removed lymph nodes were negative for tumour.

**Figure 3:**
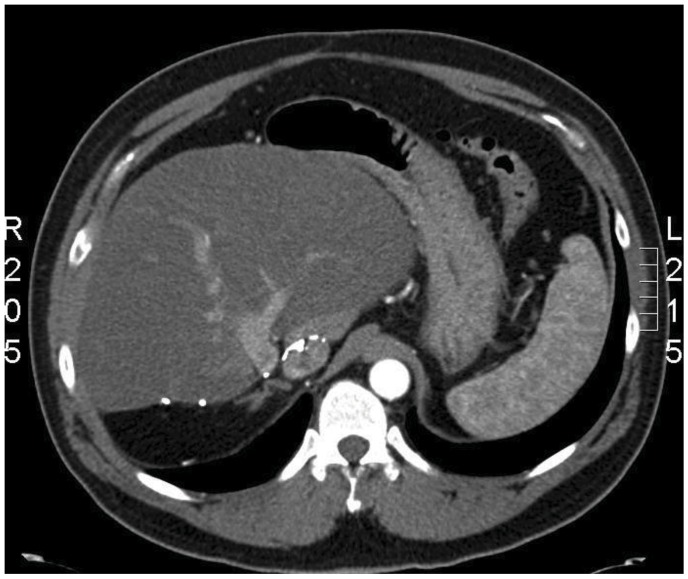
An enhanced CT scan of the abdomen 5 years following surgery demonstrates no evidence of recurrent disease in the liver.

Three months post-operatively, a FSG level, provocative secretin-stimulation test and a serum Chromgranin A were all within normal limits. Despite this, to the patient was closely followed with the potential expectation of an occult primary tumour becoming evident as time progressed. Six years following his hepatic resection the patient remains symptom free and off all medication. Both basal fasting gastrin and secretin-stimulation tests remain normal, as does his cross-sectional imaging (Figure 3).

## 3. Discussion

The diagnosis of ZES is based on the clinical endocrinopathy and confirmed by an elevated FSG level in the presence of raised gastric acid secretion. When the results of FSG and acid secretory studies are borderline or non-diagnostic, provocative testing with either intravenous secretin (2 mg/kg) or calcium or both is indicated [28]. Our patient had both an appropriate clinical setting and biochemical testing to confirm ZES. In the reported cases of primary hepatic gastrinoma 23/26 cases had appropriate biochemical diagnosis. Case #18 does not meet the clinical criteria for the diagnosis, as the patient was diagnosed at autopsy [24]. The diagnosis was based on the autopsy result demonstrating staining of the hepatic NET for gastrin. No history consistent with ZES or pre-morbid biochemical testing was reported [24]. Two additional cases (#24, #25) were diagnosed post-operatively following hepatic resections for presumed/potential malignant liver tumours [6,17]. Both patients’ tumours demonstrated positive staining for gastrin. Neither had pre-operative serum gastrin levels and only one of the two cases reported a clinical syndrome consistent with a functional gastrinoma. Following resection of the liver tumour, resolution of symptoms and a normal fasting serum gastrin were noted in the patient with a clinical syndrome suggestive of ZES (#24) [17]. It has been demonstrated that the immunohistochemical staining profile of neuroendocrine tumours does not always correlate with the clinical endocrinopathy [29]. As such, of these three cases, only case #24 is suggestive of a PHG.

Approximately 90% of gastrinomas are located in the Gastrinoma Triangle mainly in the duodenum or pancreas. Pancreatic gastrinomas tend to be relatively large, with a mean reported diameter of between 2.7 and 3.2 cm [1]. In contrast, 49%–80% of duodenal gastrinomas are less than 1 cm [1,30,31]. This smaller size makes the visualization of duodenal gastrinomas by pre-operative radiological studies significantly more problematic. Successful preoperative localization rates of traditional cross section imaging modalities including ultrasonography (US), CT, MRI, and arteriography have been reported between 20%–60% [1]. Somatostatin receptor scintigraphy (SRS) may visualize these NETs when cross sectional imaging is negative. However, the sensitivity of this modality depends on the size of the tumour, While sensitivity may be as high as 96% for NETs larger than 2 cm it is closer to 30% for those smaller than 1 cm [1]. Similarly, endoscopic ultrasound, while successful for the identification of larger duodenal and pancreatic tumours, has limited sensitivity for smaller, more difficult to localize tumours [1]. The arterial secretin injection (SASI) test may contribute to the anatomic localization of the primary tumour in the pancreas and duodenum. Unfortunately, this test is only capable to “regionalizing” the tumour to a specific arterial supply bed and cannot, on its own accurately guide surgical resection. As a result of the limited success of pre-operative localization, metastatic disease may mimic a primary tumour as the size of a duodenal primary is not strongly linked to its malignant potential. Of these 27 cases of PHG, 23 including the present case described a reasonable approach to pre-operative localization [4,7,8,9,10,11,12,13,14,15,16,17,18,19,20,21,22,25,26,27]. Of the four remaining cases, one was diagnosed at autopsy (#18), the second was diagnosed after removal of a presumed malignant liver tumour (#6), the third underwent an apparently blind exploration following the biochemical diagnosis (#23) and the fourth only described a preoperative CT scan (#15) [3,6,23,24].

Ultimately, preoperative studies will fail to localize the tumor in a significant percentage of patients, leaving the surgical exploration to be the localizing procedure. The issue of what comprises an adequate operative exploration for sporadic gastrinoma has been a subject of some debate in the literature. As the vast majority of tumours are known to be located in the duodenum and pancreas, a careful examination of these sites is mandatory. Examination of the pancreas is facilitated by mobilization of the pancreatic head (via the Kocher maneuver), body and tail to allow for bimanual palpation. The use of intraoperative ultrasound has been shown to improve the ability to locate these tumors [31]. The duodenal gastrinomas are typically small and located in the submucosa. Examination of the duodenum may be aided by intraoperative endoscopy and transillumination (TI) of the duodenal wall. However, given that many of these tumors are 2 mm or smaller, duodentomy with careful palpation is absolutely necessary in these patients. In a series of 143 patients undergoing operative exploration of occult gastrinoma, Norton *et al*. noted that the rate of biochemical cure was significantly higher when a duodentomy was performed compared to those that did not (90% *vs*. 50%) [30]. Proye *et al*. described the use of intra-operative gastrin measurement to assess completeness of resection following exploration on 20 patients with gastrinomas [32]. This technique although found to be 94% accurate in assessing whether all functioning gastrin-producing tissue has been removed, was only utilized in cases #12 and #22. Despite the reassuring results following liver resection, patient #12 went on to demonstrate biochemical evidence of residual disease at five years [20].

Our patient underwent a thorough operative exploration including mobilization of the pancreas and duodenum, bimanual palpation, intra-operative ultrasound and a duodenotomy. Of the additional 26 cases reported in the literature, 21 described an intra-operative approach to attempt localization of a primary within the gastrinoma triangle involving various combinations of intra-operative ultrasound, palpation, lymph node biopsies and endoscopic duodenal trans-illumination (Table 1). No case described the performance of a duodenotomy although this was presumed to have been performed in cases #6, #7, #8, as these cases were reported by a centre that had previously published on the importance of this manoeuvre [18,31]. Given the common scenario of an occult duodenal primary and the existing evidence supporting the necessity of performing a duodenotomy to find many of these small primary tumours, it is difficult to confidently confirm that an occult duodenal primary was thoroughly excluded in the majority of these cases (Table 1).

Gastrinoma is typically a slow-growing tumour. Consequently; a missed, occult primary tumour may not become evident for many years after surgical exploration. Given the rarity of PHG insight into the natural history must be taken from a similar clinical scenario, that of potential primary lymph node gastrinomas. In this literature, a potential lymph node primary tumour is defined when no additional site of tumour can be found at exploration and the operation results in resolution of clinical symptoms and normalization of biochemical indices. In a series of apparently primary lymph node gastrinoma, eight of the 26 presumed primary lymph node gastrinomas showed biochemical or clinical evidence of recurrence [5]. Four of the eight patients underwent a repeat exploration and a missed duodenal primary was found in three. Interestingly, the median time to recurrence in these patients was 5 years. This data provides two important pieces of information to the discussion of potential PHG. Firstly, the removal of a metastatic source can result in normalization of symptoms and biochemical indices in the early post-operative period. Secondly recurrence, suggesting a missed primary can occur after a significant period time following the initial operation. As such, PHG can only be firmly established after long-term clinical and biochemical follow-up.

Twenty-three of the 26 previously reported cases had initial clinical and biochemical evidence of cure. Median follow-up in these cases was 24 months (range 0–240). Only seven cases out of 26 reported follow-up of more than 5 years [6,14,18,20,27]. Recurrence was seen in four patients [8,14,18,20]. Two of these were local recurrences in the liver (#8), one of which reported multiple liver lesions (#22) [14,18]. One recurrence was seen in the lymph node along the lesser curvature of the stomach (#13) and the fourth was a biochemical recurrence (#12) [8,20]. A slight elevation in FSG following liver resection was reported in case #14. No anatomical evidence of either primary or recurrent disease was documented at 36 months, yet one has to question the diagnosis of PHG in this case [13]. There were two addition reports in the literature claiming the diagnosis of PHG, yet both had anatomical evidence of disease and a significant elevation of FSG levels post-operatively. As such neither of these cases were included in this report. Both secretin-stimulation and FSG determinations are necessary for the diagnosis of recurrent or persistent disease [33]. Fishbeyn *et al*. found that combination of both these tests at 2 years following surgical resection was 100% predictive disease-free status at 3 years [33]. Provocative testing was the first biochemical evidence of recurrence in 45% of the patients, whereas FSG was elevated first in only 36% of patients with recurrent disease. Long-term follow-up biochemically and radiologically are mandatory even after apparently curative resections before the diagnosis of PHG can be made with any degree of certainty.

Commonly reported cure rates for gastrinoma are around 26% for sporadic disease and typically much lower in gastrinomas associated with MEN 1 [1]. The low cure rate seen in MEN 1 is thought to reflect the presence of a field defect that leads to multiple primary tumours, which are difficult to completely eradicate with surgical therapy. Given this reality, the diagnosis of a PHG is not possible in the setting of MEN 1. Patient #10 was a confirmed case of MEN 1 and underwent at the time of his liver resection, a Whipple procedure for a pancreatic tumor that failed to stain for gastrin [16]. It is likely that the liver lesion was metastatic disease from an occult duodenal primary. In addition, patients #4, #13, #14, #17, #21 were below the age of 13 at diagnosis increasing the index of suspicion for hereditary disease [8,11,13,15,26]. Long-term follow-up and genetic testing would be required on these patients before one could confidently establish the diagnosis of PHG.

## 4. Conclusions

Primary hepatic gastrinoma causing ZES is extremely rare, yet our case illustrates that it does occur. A firm diagnosis of this rare clinical finding is complicated by the commonality of small occult duodenal primary tumors and their slow growing nature. Strong support for this rare diagnosis requires: (1) clear clinical and biochemical evidence of ZES; (2) an appropriate pre and intra-operative search for an occult primary tumor, especially the duodenum; and (3) long-term clinical, biochemical and radiologic follow-up. Although the current literature claims to include an additional 26 cases of PHG, without a thorough search for the primary and long-term follow-up data including provocative testing, we challenge several of the cases claiming such a diagnosis.
